# Pathogenesis and current therapies for non-infectious uveitis

**DOI:** 10.1007/s10238-022-00954-6

**Published:** 2022-11-24

**Authors:** Xue Wu, Mengying Tao, Ling Zhu, Ting Zhang, Ming Zhang

**Affiliations:** 1 Department of Ophthalmology, West China Hospital, Sichuan University, Chengdu, 610041 China; 2 Save Sight Institute, Faculty of Medicine and Health, The University of Sydney, Sydney, NSW 2000 Australia

**Keywords:** Uveitis, Non-infectious uveitis, Intraocular implant, Immunosuppressants, Biologic agents

## Abstract

Non-infectious uveitis (NIU) is a disorder with various etiologies and is characterized by eye inflammation, mainly affecting people of working age. An accurate diagnosis of NIU is crucial for appropriate therapy. The aim of therapy is to improve vision, relieve ocular inflammation, prevent relapse, and avoid treatment side effects. At present, corticosteroids are the mainstay of topical or systemic therapy. However, repeated injections are required for the treatment of chronic NIU. Recently, new drug delivery systems that may ensure intraocular delivery of therapeutic drug levels have been highlighted. Furthermore, with the development of immunosuppressants and biologics, specific therapies can be selected based on the needs of each patient. Immunosuppressants used in the treatment of NIU include calcineurin inhibitors and antimetabolites. However, systemic immunosuppressive therapy itself is associated with adverse effects due to the inhibition of immune function. In patients with refractory NIU or those who cannot tolerate corticosteroids and immunosuppressors, biologics have emerged as alternative treatments. Thus, to improve the prognosis of patients with NIU, NIU should be managed with different drugs according to the response to treatment and possible side effects.

## Introduction

**Fig. 1:**
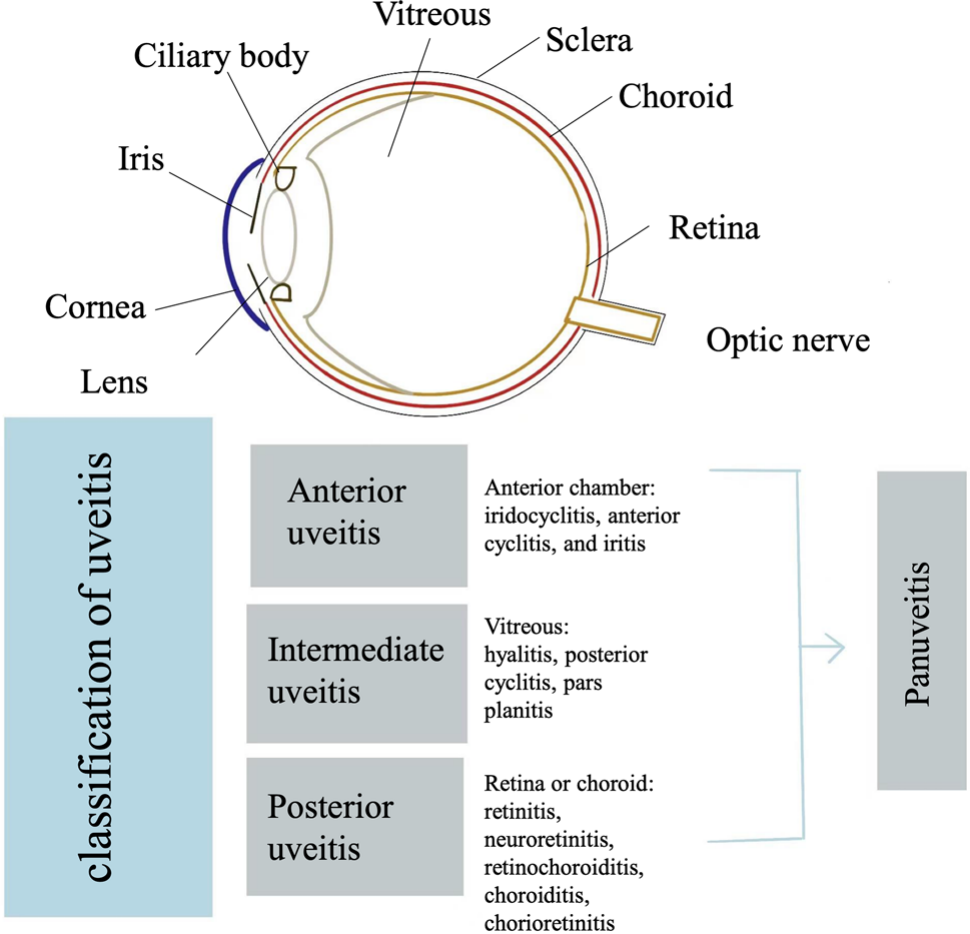
Classification of uveitis based on anatomic sites

Uveitis is a common type of ocular inflammation with various etiologies [1] that mainly affects people of working age, and uveitis results in visual impairment in up to 10% of patients of the working-age population [2]. Uveitis can be broadly classified as NIU and infectious uveitis [3]. According to the anatomical site of inflammation, NIU is further identified as anterior NIU, intermediate NIU, posterior NIU and panuveitis (Fig. 1) [3, 4]. Anterior uveitis is an inflammatory disease of the ciliary body and iris that can be diagnosed and managed early. Intermediate uveitis is associated with vitreous inflammation. Posterior uveitis involves the retina and choroid [4], and it is more sight-threatening and challenging to treat than other types of uveitis. In addition, the inflammatory lesion of NIU without specific cause and systemic diseases can only involve the eye, or it can be related to systemic autoimmune disorders, including Behcet’s disease, rheumatic diseases, and sarcoidosis [5].

The complications of uveitis include cataracts and cystic macular edema, which result from inflammation and contribute to vision loss [6–8]. Thus, NIU therapy requires the stepwise administration of anti-inflammatory drugs to relieve intraocular inflammation. Corticosteroids are reported to restrict inflammation and are considered the first-line therapy for NIU [9]. However, systemic corticosteroids are associated with dose- and duration-related adverse effects, such as secondary infections, myopathy, and hyperglycemia [10]. Furthermore, most drugs cannot be delivered directly to inflammatory lesions because of the blood–retinal barrier [11]. Therefore, intraocular injection of therapeutic drugs has been suggested to bypass this problem and reduce the adverse effects of systemic corticosteroids [11–13]. However, repeated intravitreal injections may result in endophthalmitis, hemorrhage, and retinal detachment [14]. Recently, new drug delivery systems that may ensure the intraocular delivery of therapeutic drug levels have been highlighted. With the progress in the development of drug delivery systems, therapy of NIU, especially non-infectious posterior uveitis (NIPU), has already been significantly improved over the past decade [10]. In addition, immunomodulatory agents and biologic response modifiers can be used in NIU therapy [15]. This review summarizes the potential etiologies of NIU, the preclinical findings from animal models, and the current and future treatments available to clinicians to manage NIU.

## Pathogenesis and experimental models of NIU

### Pathogenesis of NIU

Although the prevalence differs in region and ethnicity, NIU is one of the main reasons for vision loss [16]. In addition, the etiology of NIU is still not fully understood. Therefore, a better exploration of the potential inflammatory mechanisms of NIU is needed to reduce ocular inflammation and administer effective treatments. Environmental factors, molecular mimicry and hereditary susceptibility are all thought to be important factors in the pathogenesis of many forms of NIU and are associated with inflammatory cytokines and T lymphocyte subsets, which can be related to both the therapy and the clinical course of NIU [17]. Recently, the pathogenesis of NIU has been greatly developed.

The blood–ocular barrier is an anatomical barrier that anatomically prevents pathogens from peripheral bloodstream into the eye and protects ocular cells and tissues that are associated with vision. Previous reports have illustrated that cells located on the inner surface of blood–ocular barrier, including retinal pigment epithelial cells, ciliary body pigment epithelial cells, iris pigment epithelial cells, and corneal endothelial cells, participate in ocular immune system [18]. Once the blood–ocular barrier is damaged, ocular immune function can protect eye by inhibiting pathogenic T cells. Furthermore, retinal pigment epithelial cells and corneal endothelial cells can convert both CD8 + T cells and CD4 + T cells into regulatory T cells (Treg cells), while iris pigment epithelial cells can convert CD8 + T cells into Treg cells [18]. Notably, Treg cells contributed to the immune-privileged status of eye [17]. Treg cells generate transforming growth factor β, the anti-inflammatory cytokine interleukin (IL)-35, and IL-10 [19–22]. Therefore, immune response that causes pathogenic autoimmunity is prevented or suppressed by Treg cells that secrete immunosuppressive cytokines [23]. Furthermore, the cells in the eye can express special proteins (CD59, CD46, FAS/FAS ligand, and TGF-β) to restrain ocular inflammation by inactivating pathogenic lymphocytes [24–26].

Despite ocular safeguards, persistent and intense inflammation can also overcome the protection mechanisms and multilayered barriers [27]. Th17 cells are involved in early activities in the pathogenesis of inflammatory disorders [28–30]. The pathogenic molecules, such as inflammatory cytokines secreted by uveitogenic Th17 cells, promote the disruption of the blood–ocular barrier, leading to accumulation of other inflammatory cells through cytokine-receptor-JAK/STAT interactions, including monocytes, Th2, and Th1 cells that exacerbate uveitis [27, 30, 31]. Furthermore, the differences in the clinical features of NIU can be associated with the diversity of antigens that trigger the inflammatory cascade. The inflammatory cascade may also be triggered by the molecular mimicry of both antigens on invading microorganisms and self-antigens [32]. In terms of polygenic and environmental influences, the imbalance between inflammatory and regulatory mechanisms of immune system is associated with the etiology of NIU [17].

### Experimental models of NIU

Among the animal models of NIU, experimental autoimmune uveitis (EAU) is the most popular NIU animal model, contributing to a better exploration of the inflammatory origin of NIU [33]. Soluble antigen (S-Ag) and interphotoreceptor retinoid-binding protein (IRBP) are the most widely used retinal autoantigens to induce animal EAU [34]. However, the effectiveness of these autoantigens in inducing EAU differs depending on the specific species of animal used. EAU may be established in various animals, such as mice, rats and primates [35]. Both S-Ag and IRBP induce EAU in rats, whereas guinea pigs develop uveitis after immunization with S-Ag but do not respond to IRBP. In contrast, mice develop EAU upon IRBP immunization but not S-Ag immunization [36, 37]. Rats are the most widely used species for EAU due to their favorable immunogenic properties and sufficient size to provide a better model for therapeutic and surgical procedures [38].

While EAU provides a good model of the inflammatory origins of uveitis, animal models cannot totally represent human NIU [39]. There are inevitably some differences between human NIU and animal EAU. For instance, NIU in humans may be associated with distinct T-cell populations, whereas T cells in EAU involve merely one kind of retinal antigen [40]. In addition, the B cells of mice are significantly different in terms of their development, phenotypes, and immunoglobulin production when compared with those of humans [41]. Furthermore, the choroid of the human eye is much thicker than that of the mouse; thus, it forms different lymphoid-like structures and facilitates the production of different antibody levels [40]. These findings suggest that EAU cannot fully represent human NIU, and further exploration is needed.

### Clinical features and differential diagnosis of NIU

The diagnosis and differential diagnosis of NIU are challenging. Patients with NIU are at risk of retinal detachment, vision loss, cataracts and glaucoma [42]. Various factors, such as environmental, geographical, and population factors, can be involved in the differential diagnosis of NIU. NIU is also thought to be a part of some systemic diseases, which should be taken into consideration in the differential diagnosis [43, 44]. The predominant site of anterior uveitis is the anterior chamber, and anterior uveitis includes anterior cyclitis, iridocyclitis and iritis (Table 1) [4]. Clinical signs and symptoms of anterior uveitis include redness, pain, blurred vision, sensitivity to light, corneal manifestations, pupil changes, synechiae of the anterior and posterior iris, and floaters [45]. Anterior uveitis-associated systemic disorders involve sarcoidosis, juvenile idiopathic arthritis (JIA), ankylosing spondylitis, Behcet’s disease, and inflammatory bowel disease (IBD) [46]. The vitreous is the predominant site of intermediate uveitis [4]. Clinical symptoms of intermediate uveitis include sensitivity to light, floaters, and blurry vision. In addition, intermediate uveitis includes hyalitis, posterior cyclitis, and pars planitis. Furthermore, multiple sclerosis and sarcoidosis are systemic inflammatory disorders associated with intermediate uveitis [47]. The retina and choroid are the main sites of posterior uveitis, which includes retinitis, neuroretinitis, retinochoroiditis, choroiditis, and chorioretinitis [4]. Clinical signs of posterior uveitis involve floaters, usually without redness or pain. Behcet’s disease, sarcoidosis, and autoimmune disorders are systemic inflammatory disorders related to posterior uveitis [46]. Panuveitis involves the retina and/or choroid, vitreous, and anterior chamber [4]. Clinical manifestations of panuveitis include redness, pain, floaters, and sensitivity to light. Vogt‒Koyanagi‒Harada (VKH) disease, sarcoidosis, Behcet’s disease and autoimmune diseases are systemic inflammatory disorders associated with panuveitis [48].

**Table 1:** Type of uveitis according to anatomical site and corresponding systemic inflammatory diseases

Type of uveitis	Predominant site of uveitis	Clinical manifestations	Corresponding systemic inflammatory diseases
Anterior uveitis	Anterior chamber: iridocyclitis, anterior cyclitis, and iritis	Redness, pain, blurred vision, sensitivity to light, corneal manifestations, pupil changes, synechiae of the anterior and posterior iris, and floaters	Sarcoidosis, JIA, ankylosing spondylitis, IBD, and Behcet’s disease
Intermediate uveitis	Vitreous: hyalitis, posterior cyclitis, and pars planitis	Sensitivity to light, floaters, and blurry vision	Multiple sclerosis and sarcoidosis
Posterior uveitis	Retina or choroid: retinitis, neuroretinitis, retinochoroiditis, choroiditis, and chorioretinitis	Floaters, usually without redness or pain	Sarcoidosis, Behcet’s disease, and autoimmune disease
Panuveitis	Choroid, retina, vitreous, and anterior chamber	Redness, pain, floaters, and sensitivity to light	Sarcoidosis, VKH disease, Behcet’s disease, and autoimmune diseases

In addition to clinical manifestations, laboratory tests can be used in the differential diagnosis of NIU [49]. The endpoints to evaluate therapeutic effects are required to be different for the different kinds of uveitis and may be unequally applicable to all diagnoses. For example, patients with JIA-related uveitis versus those with pars planitis require different endpoints [32]. Therefore, especially close cooperation between rheumatologists and ophthalmologists in the differential diagnosis is crucial for administering appropriate therapies for patients with systemic disorders, which may benefit the long-term outcomes of these patients [45].

### Disease management

In general, the therapeutic advances in the treatment of patients with NIU include (1) systemic therapy in patients with severe NIU, (2) sustained-release corticosteroid implants, (3) systemic immunomodulators, and (4) biologic agents [50, 51]. The specific therapy administered depends on the clinical course of NIU. Short-term treatment is more aggressive for patients with acute uveitis, and a high dose of corticosteroids is needed. In addition, for patients with chronic or recurrent uveitis, a therapeutic plan to control inflammation by using a lower drug dose to reduce adverse events needs to be established [52].

### Systemic corticosteroids

Systemic therapy includes both oral and intravenous administration. Before initiating systemic corticosteroids, infectious causes must be ruled out. In addition, it is necessary to assess patients for systemic contraindications for the usage of corticosteroids before initiating therapy. Oral prednisone or prednisolone therapy is initiated at a dosage of approximately 1 mg/kg/day, which is tapered off as inflammation resolves (Table 2) [53]. The dosage of prednisone is recommended to be decreased to no more than 10 mg/day (otherwise the equivalent of other corticosteroids) [4]. Furthermore, the maximum adult dose is approximately 60–80 mg/day. No dose reduction is required if the patient has received systemic therapy with corticosteroids for less than 1–2 weeks [54]. In addition, the exact amount of prednisone reduction depends on the initial dosage: when the initial dosage is increased by 2 times, the prednisone adjustment should be reduced by approximately 2 times every 7–14 days (Table 2) [53, 55]. For rapid control of severe inflammation, including optic neuritis, serpiginous choroiditis, sympathetic ophthalmia, VKH disease, Behcet’s disease and necrotizing scleritis, pulsed intravenous treatment is recommended [54]. For vision-threatening NIU, intravenous pulse therapy of 250–1000 mg/day methylprednisolone for three consecutive days is recommended [56]. Although some adverse effects may be reversible or controllable, systemic corticosteroid treatment may be related to a risk of side effects, including adrenal suppression, osteoporosis, cushingoid changes and diabetes mellitus [55, 57].

**Table 2:** Clinical oral dosage of prednisone

Maintenance dosage	< 10 mg/day
Initial dosage	Approximately, 1 mg/kg daily
Maximum dosage of adult	60–80 mg daily
Tapering schedule	Tapering by 1–2.5 mg daily every 7–28 days for the usage of 0–10 mg/day
	Tapering by 2.5 mg daily every 7–14 days for the usage of 10–20 mg/day
	Tapering by 5 mg daily every 7–14 days for the usage of 20–40 mg/day
	Tapering by 10 mg daily every 7–14 days for the usage of over 40 mg/day
Side effects	Adrenal suppression, osteoporosis, cushingoid changes and diabetes mellitus

### New corticosteroid drug delivery systems

Recently, the following intraocular implants have been shown to decrease the frequency of injections and suppress intraocular inflammation (Table 3) [58]: (1) 0.7 mg dexamethasone implants (Ozurdex, Allergan, Irvine, California); (2) 0.19 mg fluocinolone acetonide (FA) implants (Iluvien, Alimera Sciences, Alpharetta, Georgia); (3) 0.59 mg FA implants (Retisert, Bausch and Lomb, Rochester, New York); and (4) 0.18 mg FA implants (Yutiq, EyePoint, Watertown, Massachusetts) [59]. These implants minimize the frequency of treatment and prevent the relapse of NIU involving the posterior segment. Herein, we summarize the current clinical knowledge about these implants.

**Table 3:** Summary of new drug delivery systems

Drug	Dosage	Administration method	Material property	Drug duration
Ozurdex	0.7 mg dexamethasone	23-gauge needle	Degradable	Up to 6 months
Iluvien	0.19 mg FA	25-gauge needle	Nonbiodegradable	Up to 36 months
Retisert	0.59 mg FA	20-gauge microvitreoretinal blade	Nonbiodegradable	Approximately 30 months
Yutiq	0.18 mg FA	25-gauge needle	Nonbiodegradable	Approximately 36 months

### Ozurdex

Ozurdex is a type of intravitreal implant used for NIU patients in whom it can improve visual acuity for up to 6 months and decrease intraocular inflammation [60]. Ozurdex, a biodegradable sustained-release implant, gradually releases drug into the vitreous cavity for approximately half a year, after which the polymer can be degraded into water and carbon dioxide [61]. Ozurdex is transconjunctivally inserted via the pars plana by a 23-gauge needle [61, 62]. Animal experiments demonstrated that the dexamethasone concentration in the vitreous cavity peaked at day 60 and began to decrease between day 60 and day 90. The dexamethasone concentration is maintained at a lower and steady level for up to 6 months [62, 63]. In September 2010, the Food and Drug Administration (FDA) approved Ozurdex for NIPU therapy [64]. One multicenter, longitudinal study (ClinicalTrials.gov number, NCT02951975) investigated the efficacy of dexamethasone implant on NIU [65]. The results demonstrated that there was significant improvement in mean central retinal thickness and visual acuity after therapy with Ozurdex. Although Ozurdex avoids the adverse effects of administering second-line immunosuppressants or systemic corticosteroids, it is related to a risk of cataract development and increased intraocular pressure (IOP) [66].

### Iluvien

Most recently, a novel intraocular sustained-release corticosteroid implant, Iluvien, has been used for the clinical therapy of uveitis. Iluvien can release FA for approximately 36 months [67] and play a vital role in decreasing intraocular inflammation. Iluvien is a small nonbiodegradable implant [62]. Iluvien is transconjunctivally inserted via the pars plana in the same manner as intravitreal injection through a 25-gauge needle, and the wound can be self-healing [61]. Iluvien maintained low concentrations of FA for at least 3 years [62, 68]. Iluvien was approved in several European countries, and the 0.19 mg injectable FA implant became an alternative to dexamethasone implants for the prevention of NIU recurrence [69–71]. One retrospective study evaluating the efficacy of Iluvien for non-infectious uveitic macular edema illustrated that systemic anti-inflammatory treatment was reduced or discontinued in most patients following Iluvien therapy [72]. Although it is difficult to directly compare Iluvien with Ozurdex implants regarding their safety and efficacy, both of them control inflammation well within the eye, and both have a favorable safety profile [3]. The major adverse events following Iluvien are steroid-related side effects, such as elevated IOP and cataracts [73–75].

### Retisert

Retisert, a nonbiodegradable FA implant, is administered by a 20-gauge microvitreoretinal blade [61, 62]. In 2005, Retisert was approved by FDA for therapy of chronic NIPU [76]. Retisert is a novel therapeutic method for chronic NIU, as it allows prolonged local release of steroids into the eye [77]. When compared with systemic therapy, Retisert can significantly decrease the recurrence of NIU, stabilize or improve visual acuity, and restrict eye inflammation [78, 79]. In the first month, FA was released by each Retisert implant at a rate of 0.6 μg/day. Subsequently, this rate was reduced to a stable rate of 0.3–0.4 μg/day for approximately 2.5 years [80]. In addition, pharmacokinetics of Retisert varies according to multiple factors, such as the permeability of polymers and the solubility of the drug [61]. In one randomized trial (ClinicalTrials.gov number, NCT00132691), the inflammation of NIU was controlled better with 0.59 mg FA implant than systemic treatment at 24 months [81]. However, the side effects related to Retisert also include cataracts and glaucoma [78, 79, 82]. Less common side effects are retinal detachment, vitreous hemorrhage, and scleral thinning over the implant [83–87]. The number of IOP-lowering drops required is much greater due to the higher dose of fluocinolone steroid released by the Retisert implant than the Iluvien implant [88].

### Yutiq

Yutiq is a nonbiodegradable insert with 0.18 mg FA, and the FDA also approved Yutiq for therapy of chronic NIPU [89]. Yutiq is inserted via the pars plana by a preloaded sterile applicator with a 25-gauge needle [90]. In addition, Yutiq can release FA over a period of approximately 3 years, potentially reducing the therapeutic burden in patients with NIU [91]. In one retrospective cohort study, the inflammation of NIU was controlled in 14 eyes (74%) after Yutiq treatment [91]. In addition, 0.18 mg YUTIQ is almost equal to Iluvien, which contains 0.19 mg FA, and both of them can release FA for approximately 3 years and are nonbiodegradable [92]. Unlike Retisert, Yutiq appears to have a more favorable profile and is administered in the outpatient room [90, 93]. While the most common adverse reactions of Yutiq also include cataract and increased IOP [89], Yutiq can deliver corticosteroids to the retina at a lower dose with fewer adverse events than Retisert [92].

### Immunosuppressive agents for NIU

Except for corticosteroid therapy, other therapeutic drugs to treat NIU include traditional immunosuppressants, such as cyclosporine, tacrolimus and antimetabolites. Furthermore, antimetabolites include methotrexate, mycophenolate mofetil (MMF) and azathioprine (AZA) (Table 4) [56].

**Table 4:** The usage and related side effects of immunosuppressants

Category	Drug	Dosage	Disease application	Side effects
Calcineurin inhibitors	Cyclosporine	2.5–5 mg/kg/day	Serpiginous choroiditis, Behcet’s disease-associated uveitis, VKH disease, birdshot retinochoroiditis, and idiopathic uveitis	Neurotoxicity, nephrotoxicity, hirsutism, gingivitis, hypertension and metabolic abnormalities
	Tacrolimus	2–3 mg twice a day	Behcet’s disease	Hypertension, hypomagnesemia, hyperkalemia, neurologic symptoms, diabetes, tremor, and chronic kidney disease
Antimetabolites	Methotrexate	7.5–25 mg/week	Sarcoidosis, Behcet’s disease and JIA	Fatigue, stomatitis, debilitating nausea, hepatotoxicity, cytopenia, and interstitial pneumonitis
	Mycophenolate mofetil	1 g twice a day	Scleritis, posterior and panuveitis	Gastrointestinal symptoms, leukopenia, lymphocytopenia, and elevated liver enzymes
	Azathioprine	1–3 mg/kg/day	Behcet’s syndrome and corticosteroid-resistant NIU	Allergic reactions, infection, elevated liver enzymes, bone marrow suppression, gastrointestinal reaction, and myelosuppression

### Cyclosporine

Cyclosporine is used as a second-line immunosuppressant. The immunosuppressive effects of cyclosporine occur through reversible inhibition of calcineurin and the prevention of inflammatory function of T cells in the peripheral circulation [94]. In 1983, cyclosporine was first used for therapy of uveitis by Nussenblatt et al. In addition, different research groups have investigated the effects of cyclosporine on serpiginous choroiditis, Behcet’s disease-associated uveitis, VKH disease, birdshot retinochoroiditis and idiopathic uveitis [95]. In the Systemic Immunosuppressive Therapy for Eye Diseases (SITE) study, cyclosporine monotherapy achieved 33.4% inflammation control at 6 months and 51.9% at 12 months among 373 patients with non-infectious ocular inflammation [96]. The recommended dosage of cyclosporine is 2.5–5 mg/kg/day [54]. Nevertheless, the usage of cyclosporine is related to adverse effects, such as neurotoxicity, hirsutism, gingivitis, hypertension and metabolic abnormalities [96, 97]. The most severe side effect of cyclosporine is nephrotoxicity [54]. However, nephrotoxicity is more likely to occur when cyclosporine is used in large doses.

### Tacrolimus

Tacrolimus, also known as FK506, is an immunosuppressive drug generated by *Streptomyces tsukubaensis* [54]. The mechanism by which tacrolimus inhibits T lymphocyte activation is similar to that of cyclosporine [54], and both tacrolimus and cyclosporine can inhibit calcineurin [55]. However, the immunosuppressive effect of tacrolimus is significantly better than that of cyclosporine [98, 99]. Tacrolimus can be administered intravenously or orally. The recommended dosage of tacrolimus is 2–3 mg twice a day [54]. Hogan et al. demonstrated the favorable cardiovascular risk and long-term efficacy of tacrolimus in therapy of uveitis [99]. Besides, Sloper et al. also illustrated the anti-inflammation efficacy of tacrolimus in the clinical study involving 6 patients with uveitis refractory to cyclosporine [100]. In addition, compared with cyclosporine, the duration of the efficacy of tacrolimus is longer; therefore, tacrolimus is the preferred calcineurin inhibitor for uveitis. Furthermore, tacrolimus is associated with fewer cardiovascular side effects than cyclosporine [56]. Several nonrandomized clinical studies illustrated the efficacy of tacrolimus in cyclosporine-refractory uveitis, including Behcet’s disease-associated uveitis [101, 102]. However, the side effect profile of tacrolimus is significantly better than that of cyclosporine, including hypertension, hypomagnesemia, hyperkalemia, neurologic symptoms, diabetes, tremor, and chronic kidney disease [103–105].

### Methotrexate

Methotrexate, a folic acid analog, can inhibit the enzyme that converts dihydrofolate to tetrahydrofolate [106]. Therefore, methotrexate can inhibit pyrimidine and purine synthesis by suppressing dihydrofolate reductase and thus restrain DNA production, by which methotrexate can play an essential anti-inflammatory role by inhibiting rapidly dividing cells, such as leukocytes [54]. The dosage of methotrexate can range from 25 mg subcutaneously to 7.5 mg orally once weekly [107]. The inflammation of 76% of patients was controlled by methotrexate [106]. In addition, the therapeutic effect of methotrexate in non-infectious ocular inflammation was investigated in the SITE cohort by Gangaputra et al. The results showed that eye inflammation was reduced in 66% of patients at 1 year and that methotrexate was effective for ocular inflammation [108]. For ocular inflammatory diseases, methotrexate is mainly applied in NIU related to sarcoidosis, JIA and Behcet’s disease [54]. The transient side effects of methotrexate treatment include debilitating nausea, stomatitis, and fatigue. In addition, the most severe effects of methotrexate include interstitial pneumonitis, cytopenia and hepatotoxicity [106, 108–110].

### Mycophenolate mofetil

MMF plays a vital role in the de novo synthesis of guanosine as a rate-limiting enzyme by suppressing inosine monophosphate dehydrogenase [111]. In addition, MMF has a high affinity for the activated lymphocyte subtype because lymphocytes are more dependent on this pathway, which leads to the inhibition of lymphocytes and reduction in inflammation [55]. For uveitis, MMF is recommended at a dose of 1 g twice daily [54, 112]. Based on the ability of MMF to inhibit inflammation, an 82% success rate of MMF in the treatment of inflammatory eye disease was reported by Thorne et al., and the usage of prednisolone was reduced to 10 mg per day, suggesting that MMF was an effective corticosteroid-sparing medication with controllable side effects, especially for scleritis, posterior, and panuveitis [110, 113]. Besides, Sobrin et al. also illustrated the efficacy of MMF in approximately half of patients who had previously failed or were unable to tolerate methotrexate therapy [114]. Gastrointestinal symptoms are the most common adverse events, including vomiting, abdominal pain, nausea, and diarrhea [54]. The less common adverse effects include elevated liver enzymes, lymphocytopenia, and leukopenia [54].

### Azathioprine

AZA is a precursor of 6-mercaptopurine and a purine analog [54]. AZA is incorporated into replicating DNA and thus interferes with the DNA replication process. AZA blocks the incorporation of purines into DNA in T cells and suppresses protein synthesis. The recommended usage of AZA for inhibiting ocular inflammation is a low dose of 1–3 mg/kg/day orally [115]. In one retrospective study, 62% of cases that were treated with AZA showed complete control of inflammation and 47% of subjects maintained corticosteroid-sparing control of inflammation at 1 year in ocular inflammatory diseases [116]. In addition, one randomized controlled trial illustrated that AZA efficiently controlled the eye disease associated with Behcet’s syndrome [117]. Besides, AZA inhibited inflammation in corticosteroid-resistant uveitis, although 42.8% of patients experienced side effects after AZA therapy and approximately 23.8% of patients discontinued the treatment [118]. Common side effects that lead to discontinuation include allergic reactions, infection, elevated liver enzymes, bone marrow suppression, and gastrointestinal reactions [55]. In addition, myelosuppression is the most severe adverse event [54].

### Anti-TNFα biologics

Tumor necrosis factor alpha (TNFα) inhibitors play a vital role in inflammatory diseases (Table 5). Various TNFα monoclonal antibodies are used for the treatment of inflammatory diseases. The anti-TNFα monoclonal antibody adalimumab can be injected subcutaneously. Other anti-TNFα monoclonal antibodies include infliximab, etanercept and golimumab. Therapy with biologics effectively reduces inflammation, especially in patients with inflammatory eye diseases who cannot tolerate corticosteroids [119].

**Table 5:** Summary of anti-TNFα drugs

Drug	Dosage	Disease application	Side effects
Adalimumab	40 mg every 14 days	NIPU, intermediate uveitis, panuveitis, and Behcet’s disease-related panuveitis	Serious infections, myocardial infarctions, malignancies, hematologic reactions
Infliximab	3–10 mg/kg	Behcet’s disease, JIA-associated uveitis, birdshot retinochoroiditis, IBD and sarcoidosis-associated uveitis	Infusion reactions and opportunistic infection
Etanercept	25 mg twice weekly	JIA-associated uveitis, Behcet's disease and pediatric NIPU	Injection-site reactions
Golimumab	50 mg monthly	JIA-associated uveitis	Injection-site reactions, infection, abnormal laboratory values, malignancy and congestive heart failure

### Adalimumab

Adalimumab can target TNFα as a human monoclonal antibody [55]. Although the pharmacokinetics of this biologic varies widely among patients, it has the significant advantages that it can be self-administered subcutaneously and is less immunogenic than infliximab [120]. In general, the dosage of adalimumab is 40 mg every 14 days [121]. Adalimumab was approved for subcutaneous injection to treat intermediate NIU, NIPU, and panuveitis in Europe in 2017, but only in patients who cannot tolerate corticosteroids and patients for whom corticosteroid treatment is contraindicated. In addition, adalimumab can be more effective than either etanercept or infliximab due to its better affinity for binding to TNFα [122]. VISUAL III (ClinicalTrials.gov number, NCT01148225), a multicenter clinical trial of 371 patients with active or inactive eye disease, demonstrated the efficacy of adalimumab in NIU [123]. In this study, most patients achieved quiescence and remained quiescent throughout the follow-up period. In addition, 66% of subjects were corticosteroid-free. Besides, adalimumab therapy was reported to reduce inflammation efficiently and was related to less frequency of treatment failure than placebo in uveitis related to active JIA with methotrexate therapy. Adalimumab in combination with methotrexate has been illustrated to be an efficient therapy in JIA-associated uveitis [124]. Furthermore, the FDA approved the usage of adalimumab for therapy of intermediate NIU, NIPU, and panuveitis [121]. Recently, adalimumab can be administered as a first-line treatment for Behcet’s disease-related panuveitis [125]. Serious adverse events, such as serious infections, myocardial infarctions, malignancies, and hematologic reactions, have also been reported [54].

### Infliximab

Infliximab can target TNFα as a chimeric monoclonal antibody [126]. Infliximab was the first TNFα inhibitor used to treat uveitis and is administered intravenously [126]. In addition, infliximab is also recommended as the first-line treatment for ocular Behcet’s disease [125]. Infliximab is effective for therapy of uveitis related to JIA at a dosage of 3–10 mg/kg [127]. In addition, infliximab is also beneficial to sarcoidosis, IBD and birdshot retinochoroiditis-associated uveitis [128–130]. In one retrospective study, Takeuchi et al. investigated the efficacy of infliximab in the Behcet’s disease-related uveitis. The results demonstrated that there was significant improvement in visual acuity in 55% of eyes following infliximab treatment [131]. However, infliximab might result in a higher incidence of adverse events than other TNFα inhibitors, mainly because of the immunogenicity of the mouse component of infliximab [126]. Common adverse events are infusion-site reactions [132]. Opportunistic infection is the most severe side effect. Furthermore, intravitreal infliximab has previously been reported to have potential immunogenic and retinotoxic effects [133]. Therefore, intravitreal injection of infliximab should be considered only when systemic administration of infliximab is contraindicated due to severe side effects [134].

### Etanercept

Etanercept, a recombinant fusion protein of the humanized TNFα receptor and IgG1 Fc region, acts as a decoy receptor to suppress TNFα [126, 135, 136]. The recommended dosage of etanercept is 25 mg twice weekly [6]. There are many reports of etanercept in therapy of refractory NIU, and etanercept has been extensively studied in JIA, Behcet's disease and pediatric NIPU [136–138]. Numerous studies using TNFα inhibitors for uveitis therapy demonstrated that etanercept was less effective in inducing remission and preventing relapses than infliximab [138–140]. In a meta-analysis, both infliximab and adalimumab showed better efficacy than etanercept [141]. According to available information, etanercept has been suggested as a second-line treatment (second to infliximab and adalimumab) for ocular inflammation [125, 139]. Besides, the most common adverse events are injection-site reactions [132].

However, the role of etanercept in NIU remains controversial [142]. Nowadays many studies have also demonstrated no benefit from this therapy [143, 144]. Baughman et al. investigated the efficacy of etanercept in cases with persistent ocular sarcoidosis despite methotrexate therapy. In this study, patients were administered with placebo or etanercept. However, the results illustrated that etanercept treatment was not related to significant improvement for most patients [145]. In addition, Foster et al. investigated the effect of etanercept on reducing recurrence of uveitis in patients with methotrexate therapy. The results showed no significant difference in relapse rate and final visual acuity between etanercept and placebo groups [146]. Paradoxically, there are also reports of occurrences of uveitis after etanercept administration. Some cases of uveitis are related to the administration of TNF inhibitors, particularly etanercept [147, 148]. In addition, Susanna et al. also found multiple data with new onset of uveitis after anti-TNF therapy, mainly after etanercept treatment [149].

### Golimumab

Golimumab is a novel fully human monoclonal antibody that targets TNFα [55]. Currently, the number of published studies using golimumab in NIU is small, and the sample sizes of these studies are relatively small [150–153]. The recommended dosage of golimumab is 50 mg monthly by subcutaneous injection [154]. Along with other biologics, this agent has recently been used in uveitis related to JIA [120]. Furthermore, golimumab is a viable drug candidate in patients refractory to treatment with other biologics [155]. In one multicenter study, 87% of refractory spondyloarthritis-related uveitis had complete remission after golimumab treatment [156]. Besides, Fabiani et al. found complete control of intraocular inflammation in the treatment of Behcet’s disease-associated uveitis after 12 months of follow-up [157]. The most common adverse events are injection-site reactions, and other side effects include infection, abnormal laboratory values, malignancy and congestive heart failure [158].

### Other biological therapies

#### IL-6 inhibitor

Tocilizumab (Actemra, Genentech Inc) is one kind of recombinant anti-IL-6 receptor monoclonal antibody [159, 160]. The effect of tocilizumab in the treatment of NIU was evaluated in one multicenter clinical trial [161]. In this study, 37 patients were administered with either 8 or 4 mg/kg of tocilizumab. The authors found that both doses significantly improved vision and reduced both central macular thickness and vitreous haze. The efficacy of tocilizumab in refractory JIA-related uveitis was also demonstrated in one retrospective study [162]. In this study, the anterior chamber cell improved in 79.2% of patients after half a year of therapy, and 76% of patients achieved complete remission after a median follow-up of one year. The efficacy of tocilizumab has also been explored in  Behcet’s disease-associated uveitis [163, 164], birdshot chorioretinopathy [165, 166], and Blau syndrome [167]. The adverse events of tocilizumab include allergic reactions, nausea, dizziness, gastrointestinal disorders, increase in serum aminotransferases, autoimmune cytopenia, and increased risk of infections [168–170]. In addition, ocular side effects include peripheral ulcerative keratitis and paradoxical inflammatory responses like uveitic flares [161, 171].

#### IL-1 inhibitor

Anakinra (Kineret, Swedish Orphan Biovitrum, Stockholm, Sweden), one kind of IL-1 receptor inhibitor, has been approved for Behcet’s disease, rheumatoid arthritis, and chronic infantile neurological cutaneous articular syndrome associated uveitis [172–175]. Reported side effect of anakinra includes injection site reaction, such as ecchymosis and erythema [176]. Canakinumab (Ilaris, Novartis, East Hanover, New Jersey, USA), an anti- IL-1β monoclonal antibody, has been approved for therapy of certain periodic fever syndromes and JIA. In addition, the efficacy of canakinumab in Blau syndrome-associated uveitis refractory to other medicine has also been illustrated [177]. Besides, both anakinra and canakinumab are able to decrease uveitic flares in Behcet’s disease-associated uveitis [178]. The side effects of canakinumab include nausea, injection site reactions, diarrhea, and upper respiratory tract infection [176].

#### IL-17 inhibitor

Secukinumab (Consentyx, Novartis, Basel, Switzerland) is an anti- IL-17 monoclonal antibody. One randomized controlled trial illustrated that intravenous administration of secukinumab is better than subcutaneous administration in inducing remission of NIU and clinical improvement [179]. Besides, high intravenous dosage seems to be the best approach for disease control, and low intravenous dosage or subcutaneous administration can be applied to maintenance therapy [180]. In addition, side effects of secukinumab include injection site erythema, cholecystitis, deep venous thrombosis, fatigue, headache, arthralgias, increased risk of infections, and reactivation of uveitis [176, 179–181].

#### IL-23 inhibitor

Guselkumab (Tremfya, Janssen, Beerse, Belgium) is an anti- IL-23 monoclonal antibody. A clinical case report demonstrated the deterioration of uveitis after guselkumab administration in a case with poorly controlled sarcoidosis-associated panuveitis [182]. In addition, ustekinumab (Stelara, Janssen, Beerse, Belgium), a monoclonal antibody, can target IL-12 and IL-23, and it has been reported to be an alternative for therapy of refractory uveitis associated with Behcet’s disease and psoriatic arthritis in several studies [183, 184]. In addition, another study STELARA (ClinicalTrials.gov number, NCT02911116) also explored the effect of ustekinumab on NIU, and ustekinumab seems to have favorable results with no serious side effects [160, 185]. The common adverse events associated with ustekinumab include gastrointestinal symptoms, dizziness, headache and flu-like symptoms [176].

#### CD-20 inhibitor

Rituximab (Rituxan, Genentech Inc) is an anti-CD-20 chimeric monoclonal antibody. There are many reports of rituximab in refractory NIU [186–188]. With regard to NIU, the efficacy of rituximab has been demonstrated in JIA, VKH and Behcet’s disease-associated refractory uveitis [189–192]. One study demonstrated that rituximab controlled the ocular inflammation in eight patients with JIA-related uveitis in whom biologics were ineffective [193]. Therefore, for various ocular inflammatory diseases, rituximab is one viable option when other therapies were ineffective. The adverse events of rituximab include herpes zoster, pneumonia, hives and flushing [190, 194]. In addition, hepatitis B virus infection should be tested before rituximab administration, because rituximab may lead to reactivation of hepatitis B virus [178].

#### Janus kinase (JAK) inhibitor

JAK signal transducers play an important role in biological activity of cytokines. Tofacitinib (Xeljanz, Pfizer Inc, New York, New York, USA) has been reported to selectively inhibit JAK1 and JAK3, and it has been approved for therapy of rheumatoid arthritis [31]. Tofacitinib is also effective in refractory JIA-associated uveitis without significant side effects [195, 196]. One clinical trial of JIA (ClinicalTrials.gov number, NCT02592434) showed that there was no active uveitis in the tofacitinib group, while two patients developed uveitis in the placebo group [31]. However, there are some risks with the use of tofacitinib, including venous thromboembolism, cardiovascular side effects, malignancy and infections [197]. Filgotinib (GLPG0634/GS-6034, Galapagos NV/Gilead, Mechelen, Belgium/Foster City, California, USA) has been reported to selectively inhibit JAK1, and it has been approved for therapy of rheumatoid arthritis in the European Union and Japan [31]. One randomized, placebo-controlled trial aimed to explore the effect of filgotinib on NIU. The results demonstrated that 200 mg filgotinib decreased the risk of uveitis flares compared to placebo and was well tolerated [198]. The side effects include upper respiratory infection, nasopharyngitis, headache, and nasopharyngitis [199].

#### Interferon (IFN)

IFNs are secretory glycoprotein cytokines that enhance immune response. Literature supports the use of IFN in refractory uveitic macular edema [200, 201], multiple sclerosis [202], and  Behcet’s disease [203, 204]. Interferon alpha (IFNα) is an immunomodulatory agent. Several studies have illustrated the effectiveness of IFNα in NIU as monotherapy or in combination with glucocorticoid or other immunosuppressants. For example, it has been demonstrated that IFNα is successful in achieving control of inflammation in 78% to 92% of cases for severe Behcet’s disease [205, 206]. Interestingly, IFNα appears to be superior to conventional immunosuppressive drugs for uveitic macular edema [207, 208]. Furthermore, in 24 cases of refractory macular edema secondary to anterior, intermediate, and posterior NIU, IFNα was evaluated as partial and complete resolution in 25 and 62.5%, respectively [209]. Besides, IFN-β can also be used in the treatment of NIU. In one clinical trial of uveitic cystoid macular edema secondary to multiple sclerosis, the IFN-β group had significant improvements in visual acuity and uveitic cystoid macular edema at 3 months [208]. The adverse events of IFN include transaminase elevations, alopecia, thrombocytopenia, leukopenia, depression, and Flu-like symptoms [142, 210]

### Alkylating agents

Alkylating agents have been reported to play an important role in interference with DNA replication [15, 211, 212]. Cyclophosphamide (Cytoxan, Roxane laboratories, Inc. Columbus, OH) is an alkylating agent that inhibits B-cell function through DNA cross-linking. In the SITE study, the inflammation was controlled within a year in 76% of patients after cyclophosphamide therapy [213]. However, cyclophosphamide has potent immunosuppressive effects and is cytotoxic to cells that are undergoing rapid division and differentiation, such as lymphocytes and macrophages [214, 215]. Chlorambucil (Leukeran, Aspen Global Pharma, Inc., Johannesburg, SA) is another alkylating agent and the mechanism of chlorambucil is similar to that of cyclophosphamide. In one study, 77% of 53 cases remained in remission after chlorambucil therapy [216]. However, alkylating agents are not frequently used because of their association with secondary malignancy, severe bone marrow suppression, infection, and sterility [15, 211, 212]. Therefore, it is strongly recommended to have enough water intake during treatment. Although both alkylating agents had severe side effects in the SITE study, there was no significant association with increased mortality [217].

## Conclusion

At present, NIU therapy presents a major challenge to ophthalmologists due to its diverse etiologies and its recurrent nature. NIU treatments have expanded to include conventional corticosteroids, immunosuppressants and biologics [50, 51]. Both topical and systemic corticosteroids have been used to restrain the inflammation of eye. Recently, new drug delivery systems that may ensure the intraocular delivery of therapeutic levels of drugs have been highlighted. With the progress in the development of drug delivery systems, therapy of NIU, especially NIPU, has changed over the past decade. Immunomodulatory agents, as corticosteroid-sparing therapies, have been demonstrated to be effective in NIU associated with systemic disease. However, immunosuppressants must be administered carefully because these drugs may be related to serious systemic adverse toxic effects, such as hepatic and hematologic side effects [53]. At present, biologics have been reported to inhibit inflammation. Adalimumab, a type of TNFα inhibitor, has been a novel drug approved for NIU therapy since corticosteroids in the 1960s [126], and its approval seems to be a major milestone in the developmental process of systemic therapy for NIU. Furthermore, successful management of NIU often requires clinicians to consider the pros and cons of every treatment and the personal circumstances of each patient. Adequate counseling about the reported complications and potential benefits of each treatment is necessary before any treatment is initiated. In addition, further studies on the side effects related to multiple routes of administration are needed.

## Abbreviations

NIU: Non-infectious uveitis
NIPU: Non-infectious posterior uveitis
IL: Interleukin
EAU: Experimental autoimmune uveitis
Treg cell: Regulatory T cell
S-Ag: The soluble antigen
IRBP: Interphotoreceptor retinoid-binding protein
JIA: Juvenile idiopathic arthritis
VKH: Vogt‒Koyanagi‒Harada
IBD: Inflammatory bowel disease
FDA: Food and Drug Administration
IOP: Intraocular pressure
FA: Fluocinolone acetonide
MMF: Mycophenolate mofetil
SITE: Systemic immunosuppressive therapy for eye diseases
AZA: Azathioprine
TNFα: Tumor necrosis factor alpha
JAK: Janus kinase
IFN: Interferon
